# The Molecular Landscape of Nitric Oxide in Ovarian Function and IVF Success: Bridging Redox Biology and Reproductive Outcomes

**DOI:** 10.3390/biomedicines13071748

**Published:** 2025-07-17

**Authors:** Diamandis Athanasiou, Charalampos Voros, Ntilay Soyhan, Georgia Panagou, Maria Sakellariou, Despoina Mavrogianni, Eleni Sivylla Bikouvaraki, George Daskalakis, Kalliopi Pappa

**Affiliations:** 1 First Department of Obstetrics and Gynecology, National and Kapodistrian University of Athens, 106 79 Athens, Greece; diamathan16@gmail.com (D.A.); depy.mavrogianni@yahoo.com (D.M.); gdaskalakis@yahoo.com (G.D.); kalliopi.pappa20@gmail.com (K.P.); 2 IVF Athens Reproduction Center V.A., 151 23 Maroussi, Greece; dilay000@hotmail.com (N.S.); zetat_@hotmail.com (G.P.); marisakellariou@gmail.com (M.S.); 3 Laboratory of Cell and Gene Therapy, Biomedical Research Foundation of the Academy of Athens Greece (BRFAA), 115 27 Athens, Greece; kalliopi.pappa21@gmail.com

**Keywords:** nitric oxide, follicular fluid, IVF, oocyte quality, embryo development, oxidative stress, steroidogenesis, biomarkers, controlled ovarian stimulation, reproductive medicine

## Abstract

**Background**: Nitric oxide (NO) is an important modulator of ovarian physiology, which contributes to angiogenesis, steroidogenesis, and redox control. The stable metabolites nitrate (NO_3_^−^) and nitrite (NO_2_^−^) may indicate real-time follicular function during IVF. **Methods**: In this prospective study, we included 89 women who underwent controlled ovarian stimulation. The Griess test was used to measure NO_2_-NO_3_ concentrations in follicular fluid collected on the day of oocyte retrieval. Non-parametric and correlation tests were used to investigate the associations between oocyte yield, maturity (MII), fertilization (2PN), embryo development, and hormone levels. **Results**: Higher NO_2_-NO_3_ levels were substantially associated with increased total oocyte count, MII oocytes (*p* = 0.014), and 2PN embryos (*p* = 0.029). This suggests a strong relationship between NO bioavailability and oocyte competence. NO_2_-NO_3_ levels showed a positive correlation with estradiol (*p* < 0.001) and progesterone (*p* < 0.001), suggesting a possible function in granulosa cell steroidogenesis. **Conclusions**: Follicular NO metabolites are candidate functional indicators for oocyte quality evaluation and intrafollicular steroidogenic activity. Their predictive value may improve customized IVF treatment, especially in individuals with complicated ovarian phenotypes such as PCOS or decreased ovarian reserve.

## 1. Introduction

Despite major advances in assisted reproductive technologies (ARTs), clinical pregnancy rates remain extremely variable and are closely associated with molecular interactions in the ovarian follicular and endometrial environment [1]. Nitric oxide (NO) and its metabolites, nitrate (NO_3_^−^) and nitrite (NO_2_^−^), play critical roles in ovarian function, follicular physiology, and endometrial receptivity [2]. NO is a highly reactive and diffusible free radical gas, produced from L-arginine by nitric oxide synthases (NOS) [3], a family of enzymes that includes three isoforms: endothelial NOS (eNOS), neuronal NOS (nNOS), and inducible NOS [4]. eNOS regulates vascular homeostasis, ovarian perfusion, and angiogenesis, ensuring proper follicular growth [5]. In hypoxic ovarian follicles, NO synthesis is frequently elevated as a compensatory mechanism to enhance angiogenesis and boost follicular oxygenation, whereas excessive NO generation under inflammatory circumstances can cause oxidative stress, affecting oocyte quality and embryonic survival [6].

Numerous endeavors have been undertaken in ART to identify biochemical and imaging biomarkers that can forecast ovarian response and oocyte quality. Researchers have examined several parameters, including blood AMH, AFC, follicular VEGF, oestradiol, progesterone, and oxidative stress indicators like MDA and ROS, with varying levels of effectiveness in predicting results. However, many of these indicators are static, illustrating the overall functioning of the body rather than the specific dynamics of the follicles, or failing to depict the temporal variations in follicular maturation. NO and its stable metabolites, NO_2_^−^ and NO_3_^−^, provide real-time insights into the oxidative and vascular conditions inside the follicular environment. Their rapid turnover, involvement in steroidogenic and angiogenic signaling, and association with granulosa cell activity suggest that nitric oxide may serve as a superior biomarker for oocyte competency.

NO generation and signaling in the ovarian environment are principally mediated via the activation of soluble guanylate cyclase (sGC), which causes an increase in cyclic guanosine monophosphate (cGMP), stimulating protein kinase G (PKG), a key downstream effector that controls arterial relaxation, follicular angiogenesis, and granulosa cell proliferation [7]. NO has also been proven to induce PI3K/Akt activation, boosting granulosa cell survival and proliferation while inhibiting apoptotic signals [8]. Additionally, it stabilizes HIF-1α, a transcription factor involved in angiogenesis, glucose metabolism, and erythropoiesis [9]. Maintaining appropriate oxygen levels promotes ovarian follicle development during the vascular preovulatory period [6]. However, NO excess can impair reproductive function, and it also has pathogenic consequences [10].

Follicular fluid NO_2_-NO_3_ concentrations over 40–50 µM have been associated with meiotic complications, cytoskeletal damage, and diminished fertilization potential, particularly in cases of endometriosis or metabolic dysfunction [11,12]. Physiological values typically range from 10 to 30 µM. This is beneficial for angiogenesis, steroidogenesis, and the well-being of granulosa cells.

The balance of NO and reactive oxygen species (ROS) equilibrium is required to sustain follicular vitality [13]. Depending on the local concentration and cellular context, NO may have an antioxidant or pro-oxidant effect [14]. Under normal conditions, NO scavenges superoxide radicals (O_2_^−^), reducing the oxidative stress inside the follicle [15].

In pathological situations like inflammation, ovarian aging, or metabolic malfunction, excess NO combines with superoxide to create peroxynitrite (ONOO^−^), a cytotoxic chemical that causes lipid peroxidation and mitochondrial damage [16]. This oxidative imbalance reduces oocyte quality, promotes apoptosis in granulosa cells, and contributes to embryo fragmentation during early development [17]. Finally, the correct management of NO levels inside the endometrium is critical for providing an ideal implantation environment, since both excessive and insufficient NO production have been associated with implantation failure, recurrent pregnancy loss (RPL), and pregnancy-related diseases such as preeclampsia [18].

This study delves into the complex roles of NO and its metabolites in female fertility and aims to establish a possible association between the microenvironmental metabolism of the oocytes.

## 2. Materials and Methods

This prospective, observational study was carried out at IVF Athens Reproduction Center V.A., Maroussi, Athens, Greece, with the agreement of the Scientific and Ethics Committee under Protocol No. EVD 0702/2022, on 18 April 2022. The study was designed in conformity with the Declaration of Helsinki’s ethical standards, and it adhered to all national and institutional regulations governing human research. Before enrolling in the study, all individuals provided written informed consent, ensuring voluntary participation and conformity to ethical principles for human subject research. All personal health information was anonymized and processed in line with the General Data Protection Regulation (GDPR) and institutional confidentiality rules.

### 2.1. Study Population

The study included women who had been diagnosed with infertility and were receiving COS as part of IVF treatment at the IVF Center Athens. Patients were recruited between October 2022 and October 2024 based on strict eligibility criteria meant to reduce confounding factors and ensure homogeneity in the trial sample. Each participant provided a detailed medical and reproductive history, and all women underwent a baseline gynecological assessment, which included transvaginal ultrasound, hormone profiling, and an evaluation of uterine and ovarian function prior to the start of ovarian stimulation.

The trial included women aged 20–42, with intact ovaries, a BMI of 18–35 kg/m^2^, and a normal uterine cavity confirmed by transvaginal ultrasonography or hysteroscopy. Women with regular menstrual cycles (25–35 days) and normal ovarian reserve measures (AMH > 1.1 ng/mL and AFC ≥ 5 follicles per ovary) were considered. Participants should be on their first or second IVF cycle, as we intend to avoid changes in ovarian response due to excessive past gonadotropin exposure, provided that the previous cycle did not satisfy the Bologna criteria for poor ovarian response. To mitigate the impact of recurrent exposure to gonadotropins on follicular physiology and nitric oxide metabolism, only women demonstrating a favourable response in a prior cycle were included. Patients with a history of one failed IVF cycle were eligible for inclusion if their previous cycle did not show a poor ovarian response as determined based on the Bologna criteria for poor responders.

Exclusion criteria were strictly enforced to eliminate variables that could interfere with the evaluation of NO metabolism and its impact on ovarian function and implantation potential such as women with severe endometriosis, patients having a history of ovarian surgery, chemotherapy, or radiotherapy, women with low ovarian reserve (AMH < 1.1 ng/mL and AFC < 5 follicles per ovary). Exclusion criteria also included uncontrolled diabetes mellitus, thyroid dysfunction (TSH > 4.5 µIU/mL), autoimmune disorders (e.g., systemic lupus erythematosus, Hashimoto’s thyroiditis, and antiphospholipid syndrome), and hypertension requiring medication, which can all impact ovarian vascularization and endometrial receptivity. Moreover, to establish a study population representative of healthy reproductive physiology, women with known fertility-impacting lifestyle variables such as active smoking (≥5 cigarettes/day), excessive alcohol use (>10 units per week), and drug abuse histories were also excluded.

All subjects had a pre-treatment hormonal evaluation, which included baseline serum follicle-stimulating hormone (FSH), luteinizing hormone (LH), estradiol (E2), progesterone (P4), and prolactin (PRL) levels. Thyroid function was evaluated by measuring thyroid-stimulating hormone (TSH) and free thyroxine (FT4) levels, while metabolic indicators such as fasting glucose and insulin levels were used to determine insulin resistance. Ultrasound scans were performed during the early follicular phase to evaluate ovarian morphology, AFC, and the presence of any anomalies, such as ovarian cysts or fibroids, that could affect endometrial receptivity. Following screening, suitable women were enrolled in the research and allocated to a controlled ovarian stimulation (COS) program tailored to their specific ovarian reserve factors. Prior to recruitment, all participants provided written informed consent, confirming their voluntary involvement in the study and comprehension of the research objectives and procedures.

### 2.2. Controlled Ovarian Stimulation Protocol

All individuals received controlled ovarian stimulation (COS) with a tailored gonadotropin-releasing hormone (GnRH) antagonist program, which is extensively used in clinical practice due to its safety, flexibility, and lower risk of ovarian hyperstimulation syndrome (OHSS). The purpose of COS was to synchronize the development of numerous follicles while maintaining physiological hormonal regulation and reducing patient burden. The procedure was meticulously customized to each patient’s ovarian reserve markers, age, BMI, and past response to stimulation, achieving a balance between efficacy and safety.

Stimulation began on the second or third day of the menstrual cycle, after ovarian quiescence was confirmed through transvaginal ultrasonography. Recombinant follicle-stimulating hormone (rFSH, e.g., Gonal-F or Puregon), highly purified human menopausal gonadotropin (HP-hMG, e.g., Menopur), or a combination of the two was given subcutaneously once a day. The initial dose of gonadotropins ranged between 150 and 300 IU, depending on baseline antral follicle count (AFC), serum anti-Müllerian hormone (AMH) levels, age, and BMI. Women with low ovarian reserve or a history of poor response were given larger gonadotropin doses, whereas normo-responders or younger women with normal reserves were given lower or standard dosages. In some circumstances, a “step-up” or “step-down” technique was used to dynamically alter the dose based on follicular growth rate and hormonal trends.

The choice between recombinant hCG (r-hCG) and GnRH agonist trigger was determined according to established clinical thresholds to ensure safety and mitigate the risk of OHSS. Patients classified as moderate or low risk—characterized by serum oestradiol (E2) levels below 3500 pg/mL, absence of excessive follicular recruitment, and normal ovarian morphology—received the standard 250 μg r-hCG trigger. Patients at elevated risk for OHSS, indicated by E2 levels exceeding 3500 pg/mL, more than 14 follicles measuring at least 11 mm, or exhibiting polycystic ovarian features, received a GnRH agonist trigger (triptorelin 0.2 mg). This decision-making process consistently adhered to institutional procedures and contemporary best clinical practices.

From stimulation day 5, patients were followed every 1–3 days with serial transvaginal ultrasonography to assess follicular development, count, and growth kinetics. At the same time, serum estradiol (E2) levels were measured to monitor granulosa cell function. To avoid a premature luteinizing hormone (LH) spike, a GnRH antagonist (e.g., cetrorelix acetate 0.25 mg/day or ganirelix acetate 0.25 mg/day) was taken daily once the leading follicle achieved a diameter of about 13–14 mm. The antagonist was administered up until the day before ovulation triggering. Ovulation was initiated when at least three follicles were ≥17 mm in mean diameter and estradiol levels were within the predicted range relative to the number of mature follicles. In most normoresponders and low-risk patients, final oocyte maturation was accomplished utilizing 250 μg of r-hCG (Ovitrelle). In patients with an increased risk of OHSS (as indicated by excessive follicular recruitment, high serum E2 levels > 3500 pg/mL, or polycystic ovarian morphology), a GnRH agonist trigger (e.g., 0.2 mg triptorelin) was used as an alternative, leveraging the endogenous LH and FSH surge to reduce OHSS risk.

Oocyte retrieval was carried out precisely 36 h after triggering, using a transvaginal ultrasound-guided aspiration procedure with minimal sedation. Each follicle was aspirated individually to obtain follicular fluid and oocytes in an aseptic setting. All procedures were carried out by experienced reproductive gynecologists who followed institutional regulations.

Post insemination with ICSI or on day 1 in cases with conventional IVF, zygotes were cultured in Sage 1-Step culture medium (Origio, REF 67010010A) in an FDA-approved next generation Embryoscope Plus^TM^ incubator (Vitrolofe, A/S, Viby, Denmark) under standard conditions (37 °C, 5% O_2_, 6% CO_2_, and 89% N_2_). Fertilization success was determined by calculating the fertilization rate, which is the proportion of MII oocytes that developed into two-pronuclear (2PN) zygotes following insemination. Embryo morphology and morphokinetics were evaluated and notated daily, either according to blastomere symmetry, fragmentation, and cell quantity status by specially trained embryologists using the new ASEBIR scoring system (2022), or taking into account the iDA Score version 2 provided by Embryo Viewer Software 7.8.2 (Vitrolife, A/S, Viby, Denmark). In cases with embryo transfer, the transfer took place either on Day 3 or Day 5, depending on embryo quality and clinical protocol. In accordance with institutional norms and patient counseling, no more than two embryos were transplanted per cycle. All patients received luteal phase support via subcutaneous injection of progesterone (e.g., Prolutex, IBSA, 25 mg daily) beginning the day of oocyte retrieval and lasting until the day of pregnancy testing. Progesterone assistance was provided until 10 weeks of gestation for those with positive β-hCG. The ultimate clinical endpoint was the clinical pregnancy rate, which was defined as the presence of an intrauterine gestational sac with fetal heart activity confirmed through transvaginal ultrasonography at six to seven weeks of gestation.

### 2.3. Sample Collection and Processing

Sample collection was thoroughly standardized to ensure that biochemical measurements were consistent and reproducible across subjects. Each patient provided two biological samples: follicular fluid (FF) during oocyte retrieval and peripheral venous blood at baseline and on the day of oocyte pickup (OPU). Follicular fluid was extracted under transvaginal ultrasound guidance during oocyte extraction, which was performed 36 h following the final oocyte maturation trigger. A single-lumen aspiration needle was utilized to collect follicular contents in a sterile environment. Individual follicles were aspirated into sterile, endotoxin-free round-bottom collection tubes without the use of a flushing medium. Blood contamination during piercing was avoided, as this can interfere with the biochemical makeup of follicular fluid, particularly measures of nitric oxide metabolites.

FF was visually evaluated immediately after aspiration, and only clear, non-hemorrhagic samples from follicles containing a mature metaphase II (MII) oocyte were used in the analysis. Mixed or bloody samples were removed to minimize skewed metabolite concentrations due to red blood cell breakdown or oxidative stress. Each follicle yielded about 1–2 mL of follicular fluid. To limit inter-follicular variability and maintain standardization, just one representative sample was selected per patient, preferably from the largest mature follicle.

Samples were centrifuged at 3000× *g* for 10 min at 4 °C to remove debris, cumulus cells, and remaining granulosa cells. The supernatant was carefully pipetted into sterile, RNase- and DNase-free microcentrifuge tubes to avoid cellular contamination that could interfere with subsequent assays. Aliquots were labeled with a unique patient identifier and kept at −80 °C in a monitored biobank freezer.

#### 2.3.1. Measurement of Nitric Oxide Metabolites

Measuring NO_3_^−^ and NO_2_^−^ is a valid method of nitric oxide generation and bioavailability in the follicular milieu, while direct quantification of NO is problematic due to its short half-life (milliseconds in vivo). In this study, nitrate and nitrite contents in follicular fluid were evaluated using a two-step enzymatic and colorimetric test based on the Griess reaction, a well-established method for NO metabolite measurement in clinical and experimental settings. The total nitric oxide metabolites (NOx = NO_3_^−^ + NO_2_^−^) were quantified using a commercially available Griess Reagent System (e.g., Promega, Cayman, or Sigma-Aldrich).

To allow for reliable quantification, standard calibration curves were developed using known sodium nitrate and sodium nitrite values. The assay demonstrated linearity over the predicted concentration range in follicular fluid (usually 1–100 µM). Blank controls and reagent-only wells were added in each plate to account for background absorbance and prevent contamination or cross-reaction. The assay’s detection range was 0.5–100 µM, with a lower limit of detection of 0.5 µM. Intra- and inter-assay coefficients of variation were less than 10%, indicating remarkable repeatability.

Each follicular fluid sample was subjected to two technical duplicates, and the final result utilized for statistical analysis was the mean of both results. Each plate had a standard calibration curve generated through the repeated dilution of sodium nitrate and nitrite. Blank and reagent-only controls were included to calibrate for background absorbance. This ensured that the test was reliable and consistent throughout the whole measurement process.

The Griess Reagent System (Promega, Catalogue #G2930) was employed for all biochemical assays. We established standard calibration curves utilizing sodium nitrite and sodium nitrate procured from Sigma-Aldrich in St. Louis, MO, USA. We utilised sterile, RNase-/DNase-free microcentrifuge tubes and endotoxin-free plasticware (Thermo Fisher Scientific, Waltham, MA, USA) for the handling of all samples. We utilized a Hettich Mikro 220R refrigerated centrifuge to centrifuge the samples, and we preserved small quantities of follicular fluid at −80 °C in a Panasonic Biomedical ultra-low freezer. Proficient reproductive medicine professionals employed GE Voluson S8 equipment with a 7.5 MHz vaginal probe to conduct transvaginal ultrasound examinations.

#### 2.3.2. Ultrasound and Endometrial Assessment

Ultrasound monitoring was important in our study since it allowed us to examine follicular growth and endometrial responsiveness during the entire ovarian stimulation cycle. All transvaginal ultrasounds were performed by skilled reproductive experts using a high-resolution ultrasound system with a 7.5 MHz vaginal probe, resulting in optimal image quality and measurement precision.

Follicular monitoring began on stimulation Day 5 or 6 and was repeated at frequent 1–3 day intervals, depending on the individual response. The main purpose was to monitor the number, size, and growth rate of antral and preovulatory follicles. At each visit, the dimensions of all visible follicles were measured in two perpendicular planes, and the mean diameter was computed. Special emphasis was placed on detecting the cohort of growing follicles, assessing their symmetry and synchronization, and determining the leading follicles that would exceed the ovulatory threshold. Follicles were termed preovulatory when they measured ≥17 mm in mean diameter. These data were utilized in conjunction with blood estradiol levels to determine the best time for ovulation triggering.

In addition to folliculometry, endometrial thickness and pattern were assessed on each scan, with a focus on the final pre-ovulatory assessment performed on the day before ovulation triggering. Endometrial thickness was measured in the sagittal midline plane of the uterus, from the basal layer of the anterior endometrial wall to the basal layer of the posterior wall, at the maximum distance, excluding the hypoechoic halo. Measurements were taken in millimeters (mm) and reported in all patients.

### 2.4. Outcome Measures

This study sought to determine whether nitric oxide (NO) metabolite concentrations in follicular fluid were connected with crucial indications of IVF effectiveness. The principal findings were on critical reproductive characteristics monitored during the assisted reproduction process. These included the total number of oocytes retrieved during oocyte pick-up (OPU), which was a clear indicator of the ovarian response to controlled stimulation. The quantity and fraction of mature oocytes extracted during the metaphase II (MII) stage were counted to determine cytoplasmic and nuclear maturity, which are both required for successful fertilization.

Exploratory analyses were conducted to examine the ratio of nitrate to nitrite concentrations (NO_3_^−^/NO_2_^−^), their association with oocyte maturity, and potential differences in follicular NO levels depending on the type of trigger used (human chorionic gonadotropin versus gonadotropin-releasing hormone agonist). These exploratory variables enabled a more detailed knowledge of how pharmacologic and physiologic components in the IVF process affect NO metabolism.

### 2.5. Statistical Analysis

Statistical analysis was performed using IBM SPSS software, version 31 to determine the connection between nitric oxide (NO) metabolite concentrations in follicular fluid and clinical, hormonal, and embryological outcomes in IVF. Before performing any inferential statistics, the Kolmogorov–Smirnov test was used to check the distribution of all continuous variables for normality. This enabled the appropriate selection of parametric or non-parametric statistical tests based on whether the data had a Gaussian distribution.

Continuous variables were represented as mean values with standard deviation (SD) for normally distributed data and medians with interquartile ranges (IQR) for skewed distributions. Categorical variables were summarized with frequencies and percentages. Comparative analyses were conducted to determine variations in clinical or laboratory parameters across subgroups stratified by NO metabolite concentrations, fertilization results, or embryo quality categories. Depending on the data distribution, the independent-samples *t*-test or the Mann–Whitney U test was used to compare two independent groups of continuous variables. For comparisons involving more than two groups, one-way ANOVA or the Kruskal–Wallis test was used as appropriate.

Correlation analysis was used to investigate the relationships between NO metabolite levels and hormonal, follicular, and embryological markers. Pearson’s correlation coefficient was used for variables with normal distributions, and Spearman’s rank correlation coefficient was used when the normality condition was broken. To determine statistical significance, the strength and direction of these relationships were recorded, as well as the related *p*-values. For categorical outcomes, such as the presence or absence of clinical pregnancy, associations with NO concentrations were investigated using the chi-square test or Fisher’s exact test, depending on predicted cell counts. In cases where confounding variables needed to be adjusted, multivariate regression analysis was used to identify independent predictors of key outcomes such as fertilization rate, embryo quality, or clinical pregnancy, but only variables with significant univariate associations were included in the models.

All statistical tests were two-tailed, with *p*-values less than 0.05 indicating statistical significance. The analysis was carried out according to the established protocol, with no imputation for missing data. The statistical analysis results were interpreted in light of biological plausibility and earlier literature to reach significant conclusions about the role of nitric oxide in ovarian physiology and IVF effectiveness. Any missing data points were removed from the analysis using pairwise deletion. Outliers were detected using boxplot examination and included unless biologically implausible or technically incorrect.

Clinical pregnancy was not considered a primary endpoint in this study. This choice was based on the fact that some patients chose embryo cryopreservation over fresh embryo transfer during the trial period, while others stopped treatment after oocyte retrieval. As a result, embryological and hormonal indicators up to the fertilization stage were chosen as surrogate endpoints for determining follicular and oocyte competence.

## 3. Results

The final analysis comprised 89 women who received controlled ovarian stimulation (COS) for in vitro fertilization (IVF) at the IVF Center Athens between October 2022 and October 2024. All subjects met the inclusion criteria and followed the entire stimulation, oocyte harvesting, and laboratory evaluation protocol. The following findings summarize the study cohort’s demographic and baseline clinical features. These findings provide a clinical framework for further investigations into the role of nitric oxide (NO) metabolite concentrations in follicular fluid and their relationship with oocyte quality, fertilization, and embryo development.

Table 1 summarizes the baseline demographic and clinical characteristics of the 89 individuals included in the final study. The study population had a mean age of 38.4 years, with a standard deviation indicating substantial variability, which is usual for an IVF group.

The ovarian response was characterized by an average retrieval of 6.6 oocytes per patient, with 5.3 reaching the metaphase II (MII) stage and 3.7 developing into two-pronuclear (2PN) embryos after fertilization. These embryological results indicate adequate oocyte competence and fertilization potential, allowing for substantial exploration into the underlying molecular correlates.

The average concentration of nitric oxide metabolites (NO_2_-NO_3_) in follicular fluid was 25,451.92 μM. This value represents a biochemical snapshot of the oxidative and vascular environment within the ovarian follicle. Given the dual significance of nitric oxide in physiological angiogenesis and pathologic oxidative stress, this measure is critical to the current study. The subsequent analyses aim to determine if variations in follicular fluid NO_2_-NO_3_ concentrations affect oocyte development, fertilization results, and embryo quality.

Understanding these relationships may help clarify the role of local redox balance in reproductive success and guide potential therapeutic therapies in assisted reproduction.

This study looked at how nitric oxide metabolite levels (NO_2_-NO_3_) in follicular fluid correlated with clinical, endocrine, and embryological parameters in IVF patients. Patients were divided into high and low NO groups based on their median NO_2_-NO_3_ concentration. The Mann–Whitney U test was used for comparisons due to the non-normal distribution of most variables. Furthermore, Pearson correlation coefficients were employed to assess linear relationships between NO levels and continuous variables of interest.

Table 2 shows that significant variations were found in important reproductive outcomes. The total number of oocytes retrieved was considerably higher in the high NO group (median: 6.0 vs. 3.5; *p* = 0.000), indicating a link between elevated follicular nitric oxide levels and an improved ovarian response. Similarly, the number of mature oocytes (MII) was considerably higher in this group (median: 5.0 vs. 3.0; *p* = 0.014), indicating better oocyte maturation. The frequency of two-pronuclear (2PN) zygotes, which indicate successful fertilization, was also significantly increased in the high NO group (median: 3.0 vs. 1.0; *p* = 0.029). These data imply that follicular nitric oxide bioavailability may benefit both oocyte competence and early embryonic development. In terms of hormonal parameters, patients with greater NO levels had significantly higher estradiol (E2) concentrations on the day of ovulation trigger (*p* = 0.000) and higher progesterone (PRG) levels. These findings support the suggested role of NO in boosting granulosa cell steroidogenic activity. Nitric oxide has been shown to influence the function of aromatase and other steroidogenic enzymes through cyclic GMP signaling pathways, and its local vasodilatory effect is likely to improve follicular perfusion, oxygenation, and nutrient delivery—all of which are important for steroid hormone synthesis and follicular health.

No statistically significant differences were found between the high and low NO groups in terms of LH and follicle diameters (*p* = 0.433 and *p* = 0.324, respectively). These non-significant data indicate that nitric oxide’s influence is primarily limited inside the follicular compartment, rather than being influenced by systemic endocrine variables or body composition.

The use of Mann–Whitney U testing and Pearson correlation analysis allowed for robust statistical evaluation of non-parametric and linear connections, respectively. NO_2_-NO_3_ levels showed strong correlations with estrogen and oocyte quantity, highlighting NO’s biological importance as a follicular quality index. Overall, these findings support a concept in which nitric oxide metabolism in the follicular fluid milieu reflects active redox signaling and vascular support, resulting in improved oocyte growth and fertilization outcomes. The data suggests that NO_2_-NO_3_ concentrations may serve as possible non-invasive biomarkers of follicular health and IVF success. Further investigation is warranted in larger, prospective populations.

**Figure 1:**
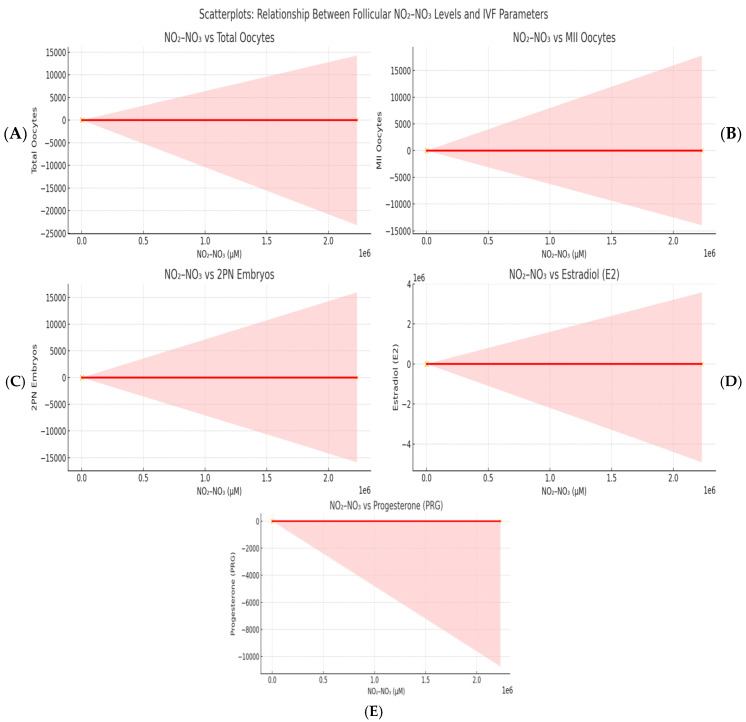
A scatterplot illustrating the correlation between follicular NO_2_-NO_3_ concentrations and significant IVF variables. Panel (**A**): Total oocyte count; (**B**): Count of MII oocytes; (**C**): Count of 2PN embryos; (**D**): Oestradiol (E2) levels on the trigger day; (**E**): Progesterone (PRG) levels. Each plot features a red line representing linear regression and a grey region indicating the 95% confidence interval. The concentrations of NO_2_-NO_3_ are measured in micromoles per litre (μM).

Figure 1 shows a significant correlation between follicular fluid NO_2_-NO_3_ concentrations and many IVF parameters. Panel A shows a positive correlation between NO_2_-NO_3_ levels and the total number of oocytes retrieved per cycle, indicating that higher intrafollicular nitric oxide activity may lead to better ovarian response. In Panel B, a similar association was seen for the number of metaphase II (MII) oocytes, implying that nitric oxide metabolites may contribute to meiotic competence and cytoplasmic maturation, both of which are important determinants of egg quality. Panel C shows a positive correlation between NO_2_-NO_3_ levels and the number of normally fertilized 2PN embryos. This study lends support to the concept that a suitable redox environment in the follicular fluid influences oocyte developmental potential and subsequent fertilization capacity. Panels D and E indicate significant positive correlations between NO_2_-NO_3_ concentrations and steroid hormone levels, specifically estradiol (E2) and progesterone (PRG), respectively. These findings are consistent with the known role of nitric oxide in boosting granulosa cell steroidogenesis, possibly through cGMP-dependent aromatase activation and enhanced follicular perfusion and oxygenation.

Because of the elective use of freeze-all techniques and patient-driven therapy termination, clinical pregnancy data were incomplete and thus not evaluated. The current study focuses on ovarian and embryological characteristics that correlate with follicular NO_2_-NO_3_ concentrations.

These findings contribute to a growing body of data showing that nitric oxide regulates folliculogenesis, meiotic spindle assembly, mitochondrial activity, and the oxidative balance required for cytoplasmic maturation. Furthermore, follicular NO_2_-NO_3_ levels were favorably linked with estradiol (E2) and progesterone (PRG) concentrations on oocyte retrieval day.

## 4. Discussion

This study sheds light on the molecular landscape of follicular fluid during controlled ovarian stimulation [19]. Elevated nitric oxide metabolite concentrations (NO_2_-NO_3_) are linked to improved ovarian performance, oocyte competence, and granulosa cell steroidogenesis [20].

Women with higher intrafollicular NO_2_-NO_3_ levels had more retrieved oocytes, MII-stage mature oocytes, and normally fertilized (2PN) embryos, as well as higher levels of estradiol (E2) and progesterone (PRG) during oocyte retrieval. These findings imply that nitric oxide, a gaseous signaling molecule well-known for its vascular functions, may also play an important role in regulating the local biochemical and cellular milieu required for optimum follicular growth and early embryogenesis [21]. Nitric oxide (NO) is enzymatically generated by three nitric oxide synthase (NOS) isoforms: neuronal NOS (nNOS), endothelial NOS (eNOS), and inducible NOS [22]. In the ovarian follicle, eNOS is expressed constitutively in the vascular endothelium and granulosa cells, although it is frequently increased in response to cytokine signaling or oxidative stress, especially in pathological conditions [23]. The balance of these isoforms and their downstream signaling cascades is crucial for controlling follicular microcirculation, oxygenation, and cell fate decisions [24].

After synthesis, NO diffuses easily across membranes and binds to soluble guanylate cyclase (sGC) in target cells, causing the creation of cyclic guanosine monophosphate (cGMP) [25]. This second messenger stimulates protein kinase G (PKG), which affects a variety of intracellular targets related to vascular tone, ion channel function, cell proliferation, mitochondrial respiration, and apoptosis regulation [26]. In the ovary, NO-mediated cGMP signaling stimulates granulosa cell proliferation, angiogenesis through elevation of vascular endothelial growth factor (VEGF), and hormone production, including aromatase-driven androgen-to-estrogen conversion [27]. This study found a positive association between follicular NO_2_-NO_3_ and E2/PRG concentrations [28]. This could be due to increased aromatase expression and LH receptor signaling in the granulosa layer, both of which are regulated by NO-cGMP pathways [29]. Furthermore, cGMP has been shown to affect intracellular calcium levels, which are required for both steroidogenic enzyme activation and follicular rupture, implying that NO plays a role in final maturation and ovulation [30].

Fertilization results (2PN embryos) showed a favorable correlation with NO_2_-NO_3_ concentrations. This can be explained by NO’s role in preserving spindle integrity, guaranteeing proper chromosomal alignment, and facilitating gap junction communication between the oocyte and surrounding cumulus cells [31]. These mechanisms are required for the synchronization of nuclear and cytoplasmic maturation and thus for effective fertilization [32]. Furthermore, NO is known to impact MAPK/ERK and PI3K/AKT signaling—two pathways that regulate oocyte survival, proliferation, and cell cycle progression—making it a critical regulator of oocyte developmental potential [33].

NO influences the transcriptional regulation of steroidogenic enzymes such as StAR (steroidogenic acute regulatory protein) and CYP11A1 (cholesterol side-chain cleavage enzyme) [34]. These interactions are critical for progesterone production, consistent with enhanced PRG levels seen in individuals with greater follicular NO_2_-NO_3_ levels. In luteinized follicles, NO may increase blood flow and cholesterol substrate availability, hence boosting corpus luteum activity and early luteal phase hormone synthesis [35].

The link between nitric oxide (NO) signaling and reproductive outcomes is becoming more widely recognized as an important component of the molecular architecture of ovarian function [36]. Our findings show that elevated levels of nitric oxide metabolites (NO_2_-NO_3_) in follicular fluid are linked to more retrieved oocytes, mature metaphase II (MII) oocytes, and normally fertilized 2PN embryos, as well as elevated intrafollicular estradiol (E2) and progesterone (PRG). These findings indicate that nitric oxide may work not only as a physiological moderator of ovarian dynamics but also as a biomarker for oocyte competency and steroidogenic activity. This viewpoint is supported by a number of clinical and experimental studies that investigate the molecular, endocrine, and vascular effects of NO in the ovarian follicle environment.

These findings align with the initial results from Anteby et al. (1996), which indicated a positive correlation between follicular nitric oxide levels, oestradiol levels, and follicular perfusion [37]. Zhao et al. (2010) demonstrated that elevated levels of follicular nitric oxide correlated with an increased number of antral follicles and enhanced clinical outcomes in IVF cycles [38]. The results, along with our findings, endorse the notion that nitric oxide may enhance follicular angiogenesis, steroidogenesis, and oocyte competence when maintained within physiological parameters. This may serve as a valuable functional biomarker during regulated ovarian stimulation.

Anteby et al. (1996) [37] were among the first to demonstrate that follicular nitrate/nitrite contents are positively linked with both follicular volume and estradiol production, implying that NO is a measure of healthy follicular expansion and granulosa cell steroidogenesis. They also found a negative association between follicular NO levels and ovarian artery resistance indices, suggesting that NO plays a role in modulating follicular perfusion and vascular tone [37]. Our findings support earlier discoveries that increased NO_2_-NO_3_ levels may enhance oxygen and nutrient supply through vasodilation, promoting granulosa cell activity and oocyte maturation [39]. Zhao et al. (2010) [38] expanded on this understanding by demonstrating that NO concentrations in follicular fluid are positively linked with antral follicle count (AFC) and ovarian volume, both of which are proven markers of ovarian responsiveness and IVF success. In their study, patients who conceived following IVF had increased NO and VEGF levels, whereas levels of endothelin-1 (ET-1), a vasoconstrictor, were lower [38]. This suggests that NO and other vasoactive chemicals interact dynamically to create a vascular milieu favorable to folliculogenesis, recruitment, and luteal transformation.

However, the role of NO is not always beneficial. NO levels can become pathologically increased in the settings of chronic inflammation or metabolic dysregulation, mostly due to the activation of inducible nitric oxide synthase (iNOS) [40]. Excess NO can react with superoxide (O_2_^−^) to generate peroxynitrite (ONOO^−^), a strong oxidant that harms lipids, DNA, and proteins [16]. Lee et al. (2000) found that patients with endometriosis and hydrosalpinx had greater NO levels in their follicular fluid, but these levels were not directly related to oocyte quality or pregnancy outcomes [11]. This shows that NO may function as a stress signal in sick follicles, with its influence on reproductive capacity being context-dependent—beneficial when balanced, deleterious when excessive [41].

Findings by Da Broi et al. (2018) [12] revealed that follicular fluid from women with mild endometriosis impaired meiotic spindle formation and nuclear maturation in bovine oocytes. Their findings lend support to the concept that altered redox and inflammatory markers in FF, such as increased NO or reactive nitrogen species, can directly damage cytoskeletal structure and chromatin stability, resulting in aberrant fertilization and poor embryo quality [12]. Basini et al. (2002) [42] discovered that tumor necrosis factor-alpha (TNF-α) decreases progesterone secretion and induces death in bovine granulosa cells, regardless of NO or cAMP signaling. In inflammatory settings, TNF-α and NO co-operate to upregulate iNOS expression in response to cytokines [42]. Thus, NO may exacerbate the impact of pro-apoptotic signals in the granulosa layer, particularly in follicles undergoing atresia or subjected to metabolic or oxidative stress.

The delicate balance between nitric oxide’s protective and harmful effects is intimately related to the oxidative stress level of the follicular environment [43]. Terao et al. (2019) [44] found that the oxidative stress index (OSI), calculated as the ratio of reactive oxygen metabolites (d-ROM) to antioxidant capacity (BAP), was inversely connected to fertilization and early embryo development. They discovered that follicles with lower oxidative stress levels produced higher-quality embryos, highlighting the importance of redox homeostasis [44]. Given that NO may both buffer oxidative stress and produce harmful radicals, its quantity within the follicle must be tightly controlled to maintain cellular function and genomic integrity.

Sun et al. (2023) [19] studied follicular fluid from women with impaired ovarian reserve (DOR) and discovered higher oxidative and inflammatory markers, including TNF-α and GSSG (oxidized glutathione), as well as decreased IL-18. These modifications had a detrimental correlation with both fertilization rates and embryo quality. Although NO was not explicitly assessed in this work, the results are consistent with the idea that follicular settings with high oxidative stress and pro-inflammatory signaling are less conducive to normal oocyte development—and that NO may act as a real-time reporter of these states [19].

Studies on polycystic ovarian syndrome (PCOS) yield similar conclusions. Niu et al. (2017) [45] discovered that PCOS patients with metabolic syndrome had increased TNF-α and reduced G-CSF levels in their follicular fluid, as well as impaired embryo quality. Their findings indicate that inflammatory cytokines modify the follicular cytokine network and may mediate the poor results observed in metabolically challenged follicles. TNF-α can increase iNOS, potentially leading to overproduction of NO and oxidative damage [45]. Liu et al. (2021) [46] confirmed the importance of oxidative stress by demonstrating that PCOS patients had greater total oxidant capacity (TOC) and malondialdehyde (MDA) levels in both FF and serum, with both indicators inversely linked with high-quality embryo development and blastocyst rates. The study found that follicular oxidative stress, rather than systemic markers, predicts IVF outcomes [46]. This is consistent with our observation that NO_2_-NO_3_ levels in FF correlate with oocyte and embryo quality.

Finally, these findings suggest that NO may not be a “one-size-fits-all” biomarker. Its interpretative value is most likely determined by its local concentration, the presence or absence of inflammation, and the follicle’s oxidative tone [47]. In physiological conditions, moderate NO levels promote follicular angiogenesis, granulosa cell proliferation, aromatase activity, and oocyte maturation [48]. Under oxidative or inflammatory stress, however, excessive NO or its byproducts may affect meiotic competence, mitochondrial function, and chromosomal integrity [49]. These findings support the notion of nitric oxide as a sensitive, context-dependent regulator of reproductive success [50]. It holds a unique position at the confluence of vascular physiology, oxidative signaling, and endocrine regulation, making it an intriguing candidate for future investigation as both a biomarker and therapeutic target in ART [51].

These connections represent nitric oxide’s possible impact on granulosa cell steroidogenesis, which is most likely mediated by the cGMP signaling cascade and vascular control in the follicular milieu [2]. This confirms previous studies reporting that NO affects aromatase activity and improves oxygen and food supply via vasodilation, resulting in robust estrogen and progesterone production.

## 5. Future Directions and Limitations

Although this study sheds new light on the role of nitric oxide metabolites in the follicular environment and their relationship with oocyte quality and IVF outcomes, several limitations and opportunities for future research must be recognized and addressed in order to solidify the clinical and biological relevance of these findings. First, the current study’s observational approach limits drawing clear causal findings. Although there were substantial connections between follicular fluid NO_2_-NO_3_ levels and critical reproductive parameters like retrieved oocytes, MII oocytes, and 2PN embryos, these interactions need to be validated through mechanistic research. Future studies could use in vitro granulosa cell culture systems, animal models, or ex vivo follicular perfusion tests to investigate the direct cellular effects of NO signaling on oocyte maturation, steroidogenesis, mitochondrial function, and meiotic competence. Interventions utilizing NOS inhibitors or inducers in these models would be especially useful for proving NO’s functional significance in human reproductive biology. Furthermore, our findings are solely predicated on surrogate endpoints and lack clinical pregnancy data; thus, subsequent research must address this gap.

Second, while the study group was well-characterized and representative of standard IVF practice, the sample size may restrict our findings’ generalizability to larger, more diverse populations. Larger, multi-center studies with stratification based on age, ovarian reserve, BMI, and infertility etiology—such as PCOS, reduced ovarian reserve (DOR), or endometriosis—are needed to study how NO_2_-NO_3_ interacts with other reproductive phenotypes. These variables may significantly regulate follicular NO production and oxidative balance, altering IVF outcomes in different ways. Quantifying total NO_2_-NO_3_ is well-established, although it only provides a glimpse of cumulative NO levels. It does not distinguish between NOS isoform activity (eNOS vs. iNOS) or cellular origin (granulosa vs. theca vs. endothelial cells), nor does it capture the real-time dynamics of NO synthesis, degradation, and downstream signaling. To address this limitation, future studies should use more sophisticated molecular tools, such as real-time NO imaging, isoform-specific expression analysis, enzyme activity assays, or compartmentalized NO metabolomics, to better capture the spatiotemporal complexity of NO signaling within the ovarian follicle.

Furthermore, the current investigation is based on a single time-point measurement—taken on the day of oocyte retrieval—and lacks information on the trajectory of NO generation during the stimulation cycle. Longitudinal studies using serial FF or serum sampling throughout folliculogenesis would help to understand the temporal link between NO signaling, hormone stimulation, vascular remodeling, and follicular health. Such investigations may also uncover important times when NO modulation could be most therapeutically useful. Future research using longitudinal follicular fluid or serum sampling at many intervals throughout the stimulation cycle would elucidate the temporal variations in nitric oxide production concerning follicular development, hormonal fluctuations, and oocyte quality over time.

From a translational standpoint, our findings pave the way for interventional trials aiming at influencing NO bioavailability. Agents including L-arginine, L-citrulline, tetrahydrobiopterin (BH4), and targeted NOS modulators could be used to boost physiological NO activity or reduce pathological overproduction. These treatments should be tried on women who have oxidative or inflammatory imbalances in their reproductive systems, such as PCOS, endometriosis, or advanced maternal age. Randomized controlled trials will be required to assess whether targeted NO modulation improves oocyte quality, fertility rate, embryo viability, and clinical pregnancy outcomes.

Standardization is an essential step toward clinical application. Pre-analytical parameters, such as sample handling, storage temperature, and centrifugation timing, impact the accuracy and repeatability of NO_2_-NO_3_ measurements in follicular fluid. Standard operating procedures and inter-laboratory calibration protocols must be developed before this biomarker can be used routinely in fertility clinics. Furthermore, normative ranges based on patient demographics and stimulation procedures must be established.

Finally, future research might look into how nitric oxide interacts with other follicular biomarkers such as AMH, VEGF, reactive oxygen species (ROS), and inflammatory cytokines in a synergistic or antagonistic manner. Multi-marker models or machine learning algorithms that incorporate NO data may improve the prediction of oocyte competency and IVF outcome, resulting in more tailored stimulation regimes and better patient counseling.

Overall, while this study provides compelling evidence for the role of nitric oxide in the ovarian follicular microenvironment, future research should focus on improving mechanistic understanding, validating clinical utility across patient populations, and developing targeted interventions to harness the physiological benefits of NO while minimizing its potential cytotoxicity under pathological conditions.

## 6. Conclusions

This study demonstrates the potential of nitric oxide metabolites in follicular fluid as dynamic indicators of oocyte quality and ovarian function in women undergoing IVF. The correlation between NO_2_-NO_3_ concentrations and key indices of oocyte competence—including MII maturation, fertilization, and early embryonic development—indicates that nitric oxide plays a crucial physiological role within the intrafollicular milieu. These findings provide compelling evidence that NO_2_-NO_3_ levels reflect the immediate functional condition of the follicle, offering unique insight into the biological viability of the oocyte.

Nitric oxide emerges as an important modulator of reproductive outcomes due to its multiple mechanisms in angiogenesis, steroidogenesis, mitochondrial control, and redox balance. Physiological NO levels appear to support important steps in folliculogenesis, including vascularization, granulosa cell activity, and oocyte cytoplasm maturation. In contrast, abnormal NO production during inflammatory or oxidative stress situations may hinder these functions, emphasizing the necessity of a well-tuned NO signaling environment. Our results show that NO is more than just a byproduct of ovarian metabolism; it also plays an active role in the molecular regulation of oocyte competence.

Our findings show that NO_2_-NO_3_ may provide real-time functional insights beyond conventional markers of ovarian reserve, such as AMH, particularly in challenging populations including women with polycystic ovary syndrome (PCOS), endometriosis, or decreased ovarian reserve. Unlike static indicators that represent long-term ovarian capacity, nitric oxide metabolites provide insight into the acute metabolic and endocrine state of individual follicles during stimulation, which may aid in the fine-tuning of clinical procedures and decision-making.

Although more mechanistic and interventional research is needed to identify the individual pathways and NOS isoforms involved, the current findings support the incorporation of nitric oxide analysis into IVF assessment frameworks. Prospective clinical trials evaluating the therapeutic modulation of nitric oxide signaling—whether through dietary supplementation, pharmacologic agents, or antioxidant strategies—may aid in the identification of tailored therapies capable of improving oocyte quality and embryo development.

It is essential to acknowledge that clinical pregnancy was not the primary objective of this investigation. Due to the discretionary nature of freeze-all procedures and cycle discontinuations, outcome data post-fertilization were not consistently available. We must exercise caution when interpreting our results. Additional prospective studies focusing on clinical pregnancy or live delivery as primary objectives are necessary to validate the use of NO_2_-NO_3_ as a biomarker in assisted reproduction.

## Figures and Tables

**Table 1:** Baseline Demographic, Hormonal, and Stimulation Characteristics of the Study Population.

Variable	Mean ± SD
Number of patients	89
Mean age (years)	38.4 ± 6.0
Mean LH (mIU/mL)	7.15 ± 10.79
Mean Estradiol (E2) (pg/mL)	1674.53 ± 1444.64
Mean NO_2_-NO_3_ (μM)	25.45 ± 23.78
Meanoocytesretrieved	6.6 ± 5.4
Mean MII oocytes	5.3 ± 4.0
Mean 2PN embryos	3.7 ± 3.6

LH (luteinizing hormone), E2 (estradiol), NO_2_-NO_3_ (sum of nitrite and nitrate concentrations in follicular fluid), MII (metaphase II oocytes), and 2PN (two-pronuclear embryos). Values are expressed as the mean ± standard deviation (SD). NO_2_-NO_3_ concentrations were quantified in micromolar (μM). Extreme outliers were eliminated following revalidation.

**Table 2:** Comparative Analysis of Clinical, Hormonal, and Embryological Variables Based on Follicular Fluid NO_2_-NO_3_ Levels.

Variable	Mean	StdDev	Min	25%	Median	75%	Max	*p*-Value
**NO_2_-NO_3_**	30.0	26.0	18.0	40.0	48.0	13.0	22.0	
**TotalOocytes**	6.76	5.0	0.0	3.5	6.0	9.0	29.0	0.0
**2PN Oocytes**	4.0	3.0	0.0	1.0	3.0	6.0	18.0	0.029
**M2 Oocytes**	5.0	4.0	0.0	3.0	5.0	8.0	19.0	0.014
**FollicleDiameter**	19.0	3.0	16.0	18.0	18.0	20.0	33.0	0.324
**PRG**	1.0	2.0	0.1	0.45	0.8	1.3	16.8	0.00
**LH**	7.0	11.0	0.5	2.32	3.55	6.15	58.0	0.433
**E2**	1675.0	1445.0	79.93	681.05	1184.0	2235.0	7315.0	0.00

Values are expressed as the mean, standard deviation (SD), minimum, 25th percentile, median, 75th percentile, and maximum. *p*-values were calculated using the Mann–Whitney U test for non-parametric group comparisons, and Pearson correlation was used for continuous variable connections. Statistically significant results (*p* < 0.05) are highlighted.

## Data Availability

The original contributions presented in this study are included in the article. Further inquiries can be directed to the corresponding author.
